# Immunoglobulin gene rearrangement in Koreans with multiple myeloma: Clonality assessment and repertoire analysis using next-generation sequencing

**DOI:** 10.1371/journal.pone.0253541

**Published:** 2021-06-24

**Authors:** Miyoung Kim, Kibum Jeon, Kasey Hutt, Alyssa M. Zlotnicki, Hyo Jung Kim, Jiwon Lee, Han-Sung Kim, Hee Jung Kang, Young Kyung Lee

**Affiliations:** 1 Department of Laboratory Medicine, Asan Medical Center, University of Ulsan College of Medicine, Seoul, South Korea; 2 Department of Laboratory Medicine, Hangang Sacred Heart Hospital, Seoul, South Korea; 3 Invivoscribe, Inc., San Diego, California, United States of America; 4 Department of Internal Medicine, Hallym University Sacred Heart Hospital, Hallym University College of Medicine, Anyang, South Korea; 5 Department of Laboratory Medicine, Green Cross Laboratories, Yongin, South Korea; 6 Department of Laboratory Medicine, Hallym University Sacred Heart Hospital, Hallym University College of Medicine, Anyang, South Korea; German Cancer Research Center (DKFZ), GERMANY

## Abstract

**Introduction:**

We assessed the applicability of next-generation sequencing (NGS)-based *IGH*/*IGK* clonality testing and analyzed the repertoire of immunoglobulin heavy chain (*IGH*) or immunoglobulin kappa light chain (*IGK*) gene usage in Korean patients with multiple myeloma (MM) for the first time.

**Methods:**

Fifty-nine bone marrow samples from 57 Korean patients with MM were analyzed, and NGS-based clonality testing that targeted the *IGH* and *IGK* genes was performed using *IGH* FR1 and *IGK* primer sets.

**Results:**

Clonal *IGH* and *IGK* rearrangements were observed in 74.2% and 67.7% of samples from Korean patients with kappa-restricted MM, respectively (90.3% had one or both), and in 60.7% and 95.5% of samples from those with lambda-restricted MM, respectively (85.7% had one or both). In total, 88.1% of samples from Koreans with MM had clonal *IGH* and/or *IGK* rearrangement. Clonal rearrangement was not significantly associated with the bone marrow plasma cells as a proportion of all BM lymphoid cells. *IGHV3-9* (11.63%) and *IGHV4-31* (9.30%) were the most frequently reported *IGHV* genes and were more common in Koreans with MM than in Western counterparts. *IGHD3-10* and *IGHD3-3* (13.95% each) were the most frequent IGHD genes; *IGHD3-3* was more common in Koreans with MM. No *IGK* rearrangement was particularly prevalent, but single *IGKV-J* rearrangements were less common in Koreans with kappa-restricted MM than in Western counterparts. *IGKV4-1* was less frequent in Koreans regardless of light chain type. Otherwise, the usages of the *IGH* V, D, and J genes and of the *IGK* gene were like those observed in previous Western studies.

**Conclusion:**

NGS-based *IGH*/*IGK* clonality testing ought to be applicable to most Koreans with MM. The overrepresentation of *IGHV3-9*, *IGHV4-31*, and *IGHD3-3* along with the underrepresentation of *IGKV4-1* and the differences in *IGK* gene rearrangement types suggest the existence of ethnicity-specific variations in this disease.

## Introduction

Myelomagenesis is a complex multistep process that may involve earlier B-cell precursors [1]. Like other types of B-lymphoid malignancies, immunoglobulin (*IG*) gene rearrangement begins at the earliest stages of B-cell development and involves recombination events between the numerous V, (D), and J genes, which result in the formation of unique V(D)J sequences that ultimately encode the antigen-binding region of the IG chain [2, 3]. Random additions or subtractions of nucleotides at the junctional regions during the process of recombination, as well as additional, subsequent changes associated with somatic hypermutation, further diversify the antigen-binding regions [4, 5]. Each rearrangement is independent and unique; as such, the probability that the same sequence would be shared by 2 unrelated cells is negligible [4–9]. This diversity of clonality constitutes the basis of currently used clonality testing strategies [4].

Clonality assessment is pivotal for the correct diagnosis of patients in whom it is difficult to distinguish multiple myeloma (MM) from non-neoplastic processes [4, 10, 11]. After treatment, residual disease can be better identified by tracking the behavior of specific clonal tumor populations [12–14]. Moreover, the measurement of minimal residual disease can predict overall and progression-free survival, which helps plan consolidation and maintenance strategies and is also useful in evaluating the comparative efficacy of novel therapies [15]. Indeed, the International Myeloma Working Group has suggested new criteria for response assessment that emphasize measuring the minimal residual disease using sensitive tools such as next-generation sequencing (NGS) for *IG* gene clonality with a high sensitivity (i.e., at least 10^−5^) [16]. However, to successfully perform clonality assessment for MM, the relevance of *IG* gene clonality testing to this disease should be assessed.

Assessment of *IG* gene clonality also provides valuable insight into the pathogenesis and natural history of lymphoid neoplasms [7]. Antigen stimulation is considered a key environmental driver of malignant transformation [7], and the class switch recombination mechanisms involved in MM-related chromosomal translocations suggest that early events in the development of this disease occur during germinal center maturation [17]. Furthermore, the presence of pre-switch B cells that are idiotypically related to malignant plasma cells has been reported [18]. Thus, evaluating the *IG* gene repertoire and identifying stereotyped clusters of *IG* receptors in patients with MM would allow deeper investigation of the role of antigen-driven stimulation in B-lymphoid tumorigenesis [7].

The incidence rate, clinical features, and outcomes of Korean patients with MM differ from those of patients belonging to other ethnicities [19–21]. The age-standardized incidence rate of MM in the United States between 1960 and 1994 was 6.3/100,000, while the associated age-adjusted mortality rate was 3.3/100,000 [20]. The corresponding rates between 1993 and 2012 for Korean patients with MM were 1.6/100,000 and 1.0/100,000, respectively [21]. Notably, the age-standardized incidence rate of MM in Korea has increased 2-fold over the past 10 years after having risen 10-fold during the preceding 20 years [22]; this may be attributable to both better detection as well as an actual increase in the number of patients with this disease in Korea [19].

The incidences of International Staging System (ISS) stages I, II, and III in Western countries are reportedly 28%, 33%, and 39%, respectively. However, an Asian study that included Korean patients found a higher incidence of advanced disease; the rates of patients with ISS stages I, II, and III were 19.9%, 36.1%, and 44%, respectively [23]. While the *IG* repertoire might be ethnicity-specific, neither this repertoire nor the applicability of NGS-based *IG* gene clonality testing has ever been reported in Asian populations with MM, including Koreans.

In this study, we aimed to better understand the repertoire of *IG* gene usage among Koreans with MM, and to explore the applicability of NGS-based *IG* gene clonality testing in clinical practice. We evaluated the status of IG heavy chain (*IGH*) and IG kappa light chain (*IGK*) gene rearrangement as well as somatic hypermutations (SHMs) in the *IGH* variable region (*IGHV*) among Korean patients with MM via NGS using the only commercially available primer sets in Korea for the first time. The *IG* gene repertoire profile and clonality detection rate in our cohort were compared with those derived in previous Western studies to gain insight into any Korean-specific features of MM.

## Materials and methods

### Patients and clinical samples

Fifty-nine cryopreserved bone marrow (BM) buffy coat samples or fresh BM aspirate from 57 patients diagnosed with MM at Hallym University Sacred Heart Hospital, Anyang, Republic of Korea between February 2007 and January 2020 were analyzed in this study. Clinical and laboratory information was obtained from electronic medical records. The study protocol was approved by the Institutional Review Board of Hallym University Sacred Heart Hospital (No. HALLYM 2018-12-034). The informed consent requirement was waived by the Institutional Review Board.

### NGS-based clonality testing targeting *IGH* and *IGK* genes

Genomic DNA (gDNA) was extracted from BM buffy coat samples by using the QIAamp DSP DNA Blood Mini QIAcube Kit (QIAGEN GmbH, Hilden, Germany) and QIAcube instrument (QIAGEN GmbH) or from BM aspirate manually using QIAamp DNA Blood Mini kit (QIAGEN GmbH) according to the manufacturer’s instructions. NGS-based clonality testing was performed using the LymphoTrack IGH FR1 assay kit A-MiSeq (Invivoscribe, Inc. San Diego, CA, USA) and LymphoTrack IGK assay kit A-MiSeq (Invivoscribe, Inc.) according to the manufacturer’s recommendations. Briefly, amplification by PCR was performed using 100 ng of gDNA per each sample, master mixes containing primers designed with barcoded sequence adaptors. After purification and quantification, libraries were sequenced on a MiSeqDx instrument (Illumina, San Diego, CA, USA) using the MiSeq Reagent Kit version 2 (500 cycles) with a length of 2×251 bp for all assays.

### NGS data analysis

The FASTQ files were analyzed using the LymphoTrack-MiSeq version 2.4.3. software (Invivoscribe, Inc.) according to the manufacturer’s guideline.

*IGH* clonal rearrangement was determined in the following situations: (i) when the total number of reads for each sample was ≥20,000 and the top merged sequence had ≥2.5% of the total reads, then the percent reads for a suspected clonal merged sequence was twice that for the third-most frequent merged sequence and the result was interpreted as clonal; and (ii) when the total number of reads for each sample was ≥10,000 but <20,000 and the top merged sequence had ≥5% of the total reads, the percent reads for a suspected clonal merged sequence was twice that for the third-most frequent merged sequence, and the result was interpreted as clonal. *IGK* clonal rearrangement was determined based on the same interpretation criteria as for the *IGH* gene.

The interpretation of the presence of IGHV SHM was as follows: (i) when there was a merged sequence showing evidence of IGH rearrangement clonality according to the above criteria, both “in-frame” and “no stop codon” values were observed, and the mutation rate within a partial V-gene was ≥2.0%, the result was interpreted as SHM being present, whereas a mutation rate to the partial V-gene of <2.0% signified the absence of SHM; and (ii) when there was no merged sequence showing evidence of *IGH* rearrangement clonality according to the above criteria or when a merged sequence showing evidence of *IGH* clonality rearrangement according to the above criteria was present, but neither an “in-frame” nor a “no stop codon” value was observed, the result was interpreted as inconclusive.

### Statistical analysis

The Mann–Whitney U-test was used to compare nonparametric quantitative variables between 2 groups. The linear regression analysis was used to investigate the relationship between 2 quantitative, continuous variables. The Fisher’s exact test was applied to compare categorical variables between 2 groups; if not applicable, the Pearson’s chi-square test was applied instead. P-values less than 0.05 were considered statistically significant. Data were analyzed using MedCalc Statistical Software version 18.9.1 (MedCalc Software bvba, Ostend, Belgium) and Microsoft Excel 2016 (Microsoft, Redmond, Washington, USA).

## Results

### Characteristics of patients and samples

The characteristics of 57 patients (59 samples) including age, sex, timing of sample collection, heavy and light chain type, risk group according to the ISS for myeloma, laboratory findings, bone lesions, and the proportion of lymphoid cells in the BM that were plasma cells are summarized in Table 1. Fifty-two patients had only 1 sample collected at the time of initial diagnosis, while 3 patients had only 1 sample collected at the time of disease monitoring (PE05 at relapse; PE12 at disease progression; PE34 at disease progression). The remaining 2 patients had both samples collected at the time of initial diagnosis and at the time of disease monitoring (relapse or disease progression status): PE10 and PE01 were from 1 patient (at the initial diagnosis and at follow-up, respectively) and PE17 and PE18 were from the other patient (at the initial diagnosis and at follow-up, respectively).

**Table 1:** Characteristics of 59 bone marrow samples from 57 Korean patients with multiple myeloma.

Characteristic	n*	No. (%) or median (range)
Sex, M/F	57	32 (56.1%) / 25 (43.9%)
Age (years)	57	70 (44–84)
Timing of sample collection, initial/follow-up (relapse)†	59	54 (91.5%) / 5 (8.5%)
Heavy and light chain type: IgG, K / IgG, L / IgA, K / IgA, L / IgD, K / IgD, L / K light chain only / L light chain only	57	17 (29.8%) / 10 (17.5%) / 10 (17.5%) / 6 (10.5%) / 0 (0.0%) / 3 (5.3%) / 2 (3.5%) / 9 (15.8%)
International Scoring System, I/II/III	57	8 (14.0%) / 17 (29.8%) / 32 (56.1%)
Beta-2 microglobulin (mg/L)	57	5.5 (1.6–22.6)
Albumin (g/dL)	57	3.4 (1.7–4.7)
Hemoglobin (mg/dL)	57	8.7 (6.5–13.9)
Creatinine (mg/dL)	57	1.10 (0.27–11.27)
Serum calcium (mg/dL)	57	8.8 (7.7–13.5)
Bone lesions, present/absent	57	44 (77.2%) / 13 (22.8%)
Proportion of plasma cells out of total lymphoid cells in bone marrow	59	75.0 (28.5–100.0)

* The number of samples was 59 when analyzing the timing of sample collection and the proportion of plasma cells among all bone marrow lymphoid cells. The number of patients from whom the samples were derived was 57.

† Two patients had both initial and follow-up samples and another three had only follow-up (relapse) samples.

### Detection rate of clonal *IGH*/*IGK* rearrangements and *IGHV* SHM

*IGH* rearrangement testing was performed in all 59 samples from 57 patients, whereas *IGK* rearrangement testing was performed in all 53 samples from 51 patients (31 samples from 29 patients with kappa-restricted MM and 22 samples from 22 patients with lambda-restricted MM) based on sample availability.

The total read counts (median [range]) for *IGH* rearrangement among the 59 samples and for *IGK* rearrangement among 53 samples were 99502 (9234–2146952) and 77399 (21148–263233), respectively. The coverage of *IGHV* as determined by the *IGHV* SHM assay (median [range]) was 99.12% (7.25–100%). The detection rates of clonal *IGH*/*IGK* rearrangements and *IGHV* SHM according to light chain restriction pattern are summarized in Table 2.

**Table 2:** *IGH* clonality, *IGH* somatic hypermutation, and *IGK* clonality according to light chain types in 59 bone marrow samples from 57 Korean patients with multiple myeloma.

Light chain type	*IGH* clonality (+)	*IGH* SHM (+) among *IGH* clonality (+) samples	*IGK* clonality (+)	Both *IGH* clonality (+) and *IGK* clonality (+)	*IGH* and/or *IGK* clonality
Kappa	23/31 (74.2%)	21/23 (91.3%)	21/31 (67.7%)	16/31 (51.6%)	28/31 (90.3%)
Lambda	17/28 (60.7%)	13/17 (76.5%)	21/22 (95.5%)	15/22 (68.2%)	24/28 (85.7%)
Total	40/59 (67.8%)	34/40 (85.0%)	42/53 (79.2%)	31/53 (58.5%)	52/59 (88.1%)

SHM, somatic hypermutation; MM, multiple myeloma.

Overall, 52 of 59 samples had clonal *IGH* and/or *IGK* rearrangements, which demonstrated an applicability rate of 88.1% when using the *IGH* FR1 assay and/or *IGK* assay in Korean patients with MM. Forty of fifty-nine had clonal *IGH* rearrangements (67.8%) and forty-two of fifty-three had clonal *IGK* rearrangements (79.2%); adding the *IGK* assay increased the positivity rate of clonal IG gene rearrangement rate from 67.8% to 88.1%. Clonal *IGH* rearrangements and clonal *IGK* rearrangements were observed in 74.2% and 67.7% of samples from patients with kappa-restricted MM, respectively, and in 60.7% and 95.5% of samples from patients with lambda-restricted MM, respectively. Overall, 90.3% and 85.7% of samples from patients with kappa-restricted MM and lambda-restricted MM, respectively, had clonal *IGH* and/or *IGK* rearrangements. Including the *IGK* assay increased the positivity rate of clonal *IG* gene rearrangement from 74.2 to 90.3% and from 60.7% to 85.7% in kappa-restricted MM and lambda-restricted MM, respectively.

Among all 40 samples exhibiting clonal *IGH* rearrangements, *IGHV* SHM was observed in 85.0%, including in 91.3% of samples from patients with kappa-restricted MM and 76.5% of samples from those with lambda-restricted MM.

### Clonal rearrangement versus the proportion of BM plasma cells

Clonal rearrangement was not associated with the BM plasma cells as a proportion of all BM lymphoid cells as estimated on the BM aspiration slides. The medians (ranges) of BM plasma cells as a proportion of all BM lymphoid cells were 76.9% (28.5–100.0%) and 67.7% (32.6–94.5%) in samples with and without clonal *IGH* rearrangement, respectively (P = 0.227). When *IGH* and *IGK* rearrangements were analyzed together, the medians (ranges) of BM plasma cells as a proportion of all BM lymphoid cells were 76.4% (28.5–100.0%) and 64.5% (32.6–92.5%) in subjects with clonal *IGH* and/or *IGK* rearrangement and in those with no rearrangement, respectively (P = 0.138). No or very weak correlation between the quantity of *IGH*/*IGK* rearrangements and the proportion of BM plasma cells as a proportion of all BM lymphoid cells was observed: y = 0.084x + 70.969 (R = 0.027, P = 0.315) in patients with *IGH* rearrangements or y = 0.231x + 59.423 (R = 0.147, P = 0.006) in patients with *IGH* and/or *IGK* rearrangements when only the larger values of the proportion of *IGH* or *IGK* rearrangements were considered.

### *IGH* rearrangement repertoire

To profile the *IGH* rearrangement repertoire, we analyzed the *VDJ* gene usage of 43 rearrangements from 39 samples with clonal *IGH* rearrangement (35 samples with single IGH rearrangement and 4 samples with double *IGH* rearrangements; a PE18 sample representing a relapse was excluded because it showed the same result as an initial PE17 sample from the same patient).

Overall, *IGHV3* was the most frequently reported *IGHV* group gene (67.44%) followed by *IGHV4* (20.93%), *IGHV1* (4.65%), *IGHV2* (4.65%), and *IGHV7* (2.33%). *IGHV5* and *IGHV6* were not observed in our sample set. Twenty *IGHV* genes were identified (Table 3); among them, *IGHV3-23* and *IGHV3-9* (each of which was observed in 11.63% of the total samples) were the most frequently reported, followed by *IGHV3-30* and *IGHV4-31* (each of which was observed in 9.30% of the total samples). Comparing *IGHV* usage between our study and 3 previously published Western studies [7, 8, 24] showed that even though the distribution of the *IGHV* gene group in our study was similar to that of Western studies (S1 Table in S2 File, S1 Fig in S1 File), the distribution of *IGHV* genes was different between ours and Western studies (S2 Table in S2 File, S2 Fig in S1 File). Particularly, *IGHV3-9* (11.63%) and *IGHV4-31* (9.30%) were significantly more frequent in our study than in that of Ferrero et al. (4.64%, P = 0.004 and 2.03%, P = 0.024, respectively) [7] and of Medina et al. (4.14%, P < 0.001 and 1.38%, P = 0.009, respectively) [24]. The overall frequencies of other genes were not significantly different across all 4 studies (including ours).

**Table 3:** The *IGHV* gene repertoire in 43 *IGH* rearrangements from 39 samples with clonal *IGH* rearrangements.

	n	%
*IGHV1*	2	4.65%
* IGHV1-2*	1	2.33%
* IGHV1-8*	1	2.33%
*IGHV2*	2	4.65%
* IGHV2-5*	2	4.65%
*IGHV3*	29	67.44%
* IGHV3-21*	4	9.30%
* IGHV3-23*	5	11.63%
* IGHV3-30*	4	9.30%
* IGHV3-30-3*	2	4.65%
* IGHV3-33*	2	4.65%
* IGHV3-48*	1	2.33%
* IGHV3-49*	1	2.33%
* IGHV3-64*	1	2.33%
* IGHV3-66*	1	2.33%
* IGHV3-7*	1	2.33%
* IGHV3-74*	2	4.65%
* IGHV3-9*	5	11.63%
*IGHV4*	9	20.93%
* IGHV4-31*	4	9.30%
* IGHV4-4*	1	2.33%
* IGHV4-59*	2	4.65%
* IGHV4-61*	2	4.65%
*IGHV5*	0	0.00%
*IGHV6*	0	0.00%
*IGHV7*	1	2.33%
* IGHV7-4-1*	1	2.33%

In the *IGHJ* group, *IGHJ4* was predominant (44.19%) followed by *IGHJ6* (25.58%), *IGHJ5* (16.28%), and *IGHJ3* (11.63%). Neither *IGHJ1* nor *IGHJ2* was reported in our patients. The *IGHJ* distribution in our study was similar to that observed in the previous Western studies [7, 8, 24] (S3 Table in S2 File, S3 Fig in S1 File).

The most frequently reported *IGHD* gene group was *IGHD3* (34.88%) followed by *IGHD2* (20.93%), *IGHD5* (9.30%), *IGHD4* and *IGHD6* (4.65% each), and *IGHD1* (2.33%). *IGHD7* was not observed in our series. Five samples (11.63%) had *D*-gene sequences that were not matched to the reference set of currently known *D*-region sequences owing to the sequences being either too short or too distinct to be matched. Another 5 samples (11.63%) did not exhibit any *D*-gene sequences. Twenty-seven *IGHD* genes were identified (Table 4), the most frequently occurring of which were *IGHD3-10* and *IGHD3-3* (13.95% each). A comparison of *IGHD* usage between our study and 3 previously published Western studies [7, 8, 24] showed that even though the distribution of the *IGHV* gene group was similar (S4 Table in S2 File, S4 Fig in S1 File), *IGHD3-3* was more frequent in our study than in that of Medina et al. (13.95% vs. 5.44%, P = 0.038) [24] (S5 Table in S2 File, S5 Fig in S1 File).

**Table 4:** The *IGHD* gene repertoire in 43 *IGH* rearrangements from 39 samples with clonal *IGH* rearrangements.

	n	%
*IGHD1*	1	2.33%
*IGHD1-14*	1	2.33%
*IGHD2*	9	20.93%
*IGHD2-15*	1	2.33%
*IGHD2-2*	4	9.30%
*IGHD2-21*	1	2.33%
*IGHD2-8*	3	6.98%
*IGHD3*	15	34.88%
*IGHD3-10*	6	13.95%
*IGHD3-16*	2	4.65%
*IGHD3-3*	6	13.95%
*IGHD3-9*	1	2.33%
*IGHD4*	2	4.65%
*IGHD4-17*	1	2.33%
*IGHD4-23*	1	2.33%
*IGHD5*	4	9.30%
*IGHD5-12*	3	6.98%
*IGHD5-5*	1	2.33%
*IGHD6*	2	4.65%
*IGHD6-13*	2	4.65%
Unknown*	5	11.63%
Not found	5	11.63%

### *IGK* rearrangement repertoire

On *IGK* rearrangement repertoire analysis, 4 rearrangements from 2 samples (PE01 and PE18, which represented relapses of PE10 and PE17, respectively) were excluded from the repertoire analysis given that each showed the same rearrangement pattern as its original pre-relapse sample. Thus, we analyzed the *V(D)J* gene usage of 85 rearrangements from 40 samples with clonal *IGK* rearrangement (10 samples with a single *IGK* rearrangement, 18 with double *IGK* rearrangements, 10 with triple *IGK* rearrangements, and 2 with 4 or 5 *IGK* rearrangements).

The distribution of *IGK* locus rearrangements is summarized in Table 5. Overall, the *IGKV-J* rearrangement was the most common (17.50%), followed by *IGKV-J* + *IGKV-KDE* and *IGKV-J* + *IGKJ-C-intron-KDE* (15.00% each). In kappa-restricted MM, the *IGKV-J* rearrangement was the most common (31.58%) followed by *IGKV-J* + *IGKV-KDE* (26.32%); each of which was observed only in 4.76% of lambda-restricted MM. In lambda-restricted MM, *IGKV-J* + *IGKJ-C-intron-KDE* and *IGKV-J + IGKV-KDE + IGKJ-C-intron-KDE* were the most common types (19.05% each). A comparison between our study and a previous Western analysis [24] showed that a lack of *IGK* rearrangement was significantly more frequent among our patients (21.57% vs. 7.06%, P = 0.017), particularly in kappa-restricted MM (34.48% vs. 0.00%, P < 0.001) (S6 Table in S2 File, S6 Fig in S1 File). Furthermore, single *IGKV-J* rearrangement was less frequent in Korean patients with kappa-restricted MM than in their Western counterparts (20.69% vs. 44.19%, P = 0.047).

**Table 5:** Distribution of 85 *IGK* locus rearrangements from 40 samples with clonal *igk* rearrangement.

	Kappa-restricted MM		Lambda-restricted MM		Total	
	n = 19	%	n = 21	%	n = 40	%
*IGKV-J*	6	31.58%	1	4.76%	7	17.50%
*IGKV-KDE*	1	5.26%	2	9.52%	3	7.50%
*IGKJ-C-intron-KDE*	0	0.00%	0	0.00%	0	0.00%
*IGKV-J + IGKV-J*	1	5.26%	2	9.52%	3	7.50%
*IGKV-J + IGKV-KDE*	5	26.32%	1	4.76%	6	15.00%
*IGKV-J + IGKJ-C-intron-KDE*	2	10.53%	4	19.05%	6	15.00%
*IGKV-KDE + IGKJ-C-intron-KDE*	0	0.00%	2	9.52%	2	5.00%
2 *IGKV-KDE*	0	0.00%	1	4.76%	1	2.50%
2 *IGKV-J + IGKJ-C-intron-KDE*	2	10.53%	0	0.00%	2	5.00%
*IGKV-J + IGKV-KDE + IGKJ-C-intron-KDE*	1	5.26%	4	19.05%	5	12.50%
2 *IGKV-KDE + IGKJ-C-intron-KDE*	0	0.00%	1	4.76%	1	2.50%
*IGKV-J +* 2 *IGKJ-C-intron-KDE*	0	0.00%	2	9.52%	2	5.00%
Others (4 or 5 rearrangements)	1	5.26%	1	4.76%	2	5.00%

*IGKV1* was the most frequently observed *IGKV* gene group (32.94%), followed by *IGKV2* (22.35%), and *IGKV3* (12.94%). *IGKV4*, *IGKV5*, and *IGKV7* were observed in a minority of the series (5.56%, 2.78%, and 2.78%, respectively). The remaining 13.89% were observed in rearrangements involving *IGKJ-C-intron-KDE*.

The most frequently observed *IGKV* gene was *IGKV2-30* (11.76%), followed by *IGKV1D-33* (10.59%) in the total series, comprising 13.89% each in kappa-restricted MM and 10.20% and 8.16% in lambda-restricted MM, respectively (Table 6). A comparison between our study and a previous Western analysis [24] showed that *IGKV4-1* was significantly less frequent in our series regardless of light chain type than in the Western study (where it was the most frequent gene): 3.53% vs. 22.54% in kappa-restricted MM (P < 0.001), 5.56% vs. 11.54% in lambda-restricted MM (P = 0.037), and 2.04% vs. 21.05% in the total series (P = 0.019) (S7 Table in S2 File, S7 Fig in S1 File).

**Table 6:** The *IGKV*(*D*) gene repertoire in 85 *igk* rearrangements from 40 samples with clonal *igk* rearrangements.

	Kappa-restricted MM		Lambda-restricted MM		Total	
	n = 36	%	n = 49	%	n = 85	%
*IGKV1-17*	1	2.78%			2	2.35%
*IGKV1-27*			3	6.12%	2	2.35%
*IGKV1-5*	2	5.56%	1	2.04%	3	3.53%
*IGKV1-9*	1	2.78%			1	1.18%
*IGKV1D-12*	1	2.78%			1	1.18%
*IGKV1D-33*	5	13.89%	4	8.16%	9	10.59%
*IGKV1D-37*	1	2.78%	2	4.08%	3	3.53%
*IGKV1D-39*	3	8.33%	3	6.12%	6	7.06%
*IGKV1D-8*			1	2.04%	1	1.18%
*IGKV2-24*	1	2.78%			1	1.18%
*IGKV2-29*			2	4.08%	2	2.35%
*IGKV2-30*	5	13.89%	5	10.20%	10	11.76%
*IGKV2D-26*	1	2.78%	1	2.04%	2	2.35%
*IGKV2D-28*	1	2.78%	1	2.04%	2	2.35%
*IGKV2D-29*			1	2.04%	1	1.18%
*IGKV2D-40*	1	2.78%			1	1.18%
*IGKV3-11*	1	2.78%	1	2.04%	2	2.35%
*IGKV3-15*	1	2.78%			1	1.18%
*IGKV3-7*			2	4.08%	2	2.35%
*IGKV3D-20*	2	5.56%	3	6.12%	5	5.88%
*IGKV3D-7*			1	2.04%	1	1.18%
*IGKV4-1*	2	5.56%	1	2.04%	3	3.53%
*IGKV5-2*	1	2.78%	1	2.04%	2	2.35%
*IGKV7-3*	1	2.78%			1	1.18%
*Intron-KDE*	5	13.89%	16	32.65%	21	24.71%

*IGKJ4* was predominant (20.00%) followed by *IGKJ2* (11.76%), *IGKJ1* (7.06%), *IGKJ3* (4.71%), and *IGKJ5* (3.53%). The remaining 52.94% were observed in rearrangements involving *IGKJ-C-intron-KDE*, *IGKV-KDE*, or rearrangements with no *J*-gene.

## Discussion

We investigated the applicability of the only commercially available NGS-based *IG* gene rearrangement test and analyzed the repertoire of *IGH* and *IGK* gene usage in Korean patients with MM at baseline for the first time. Clonal *IGH* and/or *IGK* rearrangements were observed in 88.1% of the samples. No significant association was observed between clonal rearrangement and the proportion of BM lymphoid cells that were plasma cells. The *IGH* and *IGK* repertoires were similar to those found in previous studies of Western patients with the following exceptions: (i) *IGHV3-9* (11.63%) and *IGHV4-31* (9.30%) were significantly more frequent in our study than in the Western studies (4.64% and 4.14% vs. 2.03% and 1.38%); (ii) *IGHD3-3* (13.95%) was more frequent in our study than in a Western study (5.44%); (iii) *IGK* rearrangements were more frequently absent in our study than in a Western series (21.57% vs. 7.06%), particularly in kappa-restricted MM (34.48% vs. 0.00%); (iv) single *IGKV-J* rearrangements were less frequent in Korean patients with kappa-restricted MM (20.69%) than in their Western counterparts (44.19%); and (v) *IGKV4-1* was significantly less frequent in our study but was the most common rearrangement in Western studies (3.53% vs. 22.54% in kappa-restricted MM, 5.56% vs. 11.54% in lambda-restricted MM, and 2.04% vs. 21.05% overall).

NGS-based *IG* gene rearrangement testing can identify clonality markers in patients with lymphoproliferative conditions, including lymphoid malignancies such as MM. This serves to distinguish such lymphoid malignancies from reactive processes [10, 11], identify any clonal relationships of recurrent lesions in the same patient [5], and monitor for any minimal residual disease [16]. NGS-based testing also has some advantages over flow cytometry in terms of the detection of clonal plasma cells as follows: (i) it does not require immediate analysis of fresh samples and can be applied to archived samples, (ii) it provides the sequence information of the clonal rearrangement, and (iii) it is barely affected by immunophenotypic changes that occur owing to chemotherapy [25]. Flow cytometry can be universally applied to almost all patients [26]. The technical feasibility of NGS-based *IG* gene rearrangement testing for a particular disease depends on the detection rate of clonal rearrangement within *IG* genes at baseline; however, this has only recently been investigated in very few studies of MM whereas it has been extensively explored in patients with acute lymphoblastic leukemia. Ours is the first study to investigate the applicability of such a test in Koreans with MM. We found that the detection rate of clonal rearrangements using the NGS-based *IGH* FR1 assay and/or *IGK* assay (LymphoTrack^®^ IGH FR1 and IGK assays) was 88.1%. The detection rates of clonal *IGH* and/or *IGK* rearrangements were similar to those from other studies even though the sizes and compositions of the study populations, the primer sets, and methodologies between them varied. In a study using the LymphoTrack^®^ IGH FR1, IGH FR2, IGH FR3, IGH leader, and IGK assays, Rustad et al. reported an overall clonality detection rate of 81% in patients with MM [9]. The detection rates of *IGH* FR1, *IGH* FR2, and *IGH* FR3 were similar (approximately 50%). They did not specify the additive values of the FR2 and FR3 primer sets [9]. In another study using the same primer sets, Arcila et al. reported an overall clonality detection rate of approximately 95% [4]. However, it was around 70% for MM but around 95% for B-acute lymphoblastic leukemia when only the *IGH* FR1 primer set was used. They claimed that the leader assay showed a significant additive value even though they did not specify it; moreover, the subsequent use of the FR2 and FR3 primers did not significantly increase the clonal detection rate in their study. Since only the *IGH* FR1 primer set was available in Korea, we were not able to compare our results directly to those of studies in which other *IGH* primer sets including *IGH* FR2 and *IGH* FR3 were tested.

We used the *IGK* primer sets to investigate whether there were any additional benefits to testing light chain gene rearrangement. Adding the *IGK* test increased the detection rate from 67.8% to 88.1% in the total series (from 74.2% to 90.3% in kappa-restricted MM and from 60.7% to 85.7% in lambda-restricted MM); illustrating that an additional benefit indeed exists. As only a few studies evaluated the usefulness of the *IGK* rearrangement test with different sets of primers, we were not able to compare our results directly to those of previous studies. Rustad et al. reported an overall clonal *IGK* rearrangement rate of 55% using the LymphoTrack^®^ *IGK* assay but did not specify the value of adding the *IGK* assay to *IGH* assays [9]. They reported *V–J* and *KDEL* rearrangements in 78% and 16% of kappa-restricted samples, respectively; these rates were 57.1% and 35.7%, respectively, in our study. Arcila et al. [4] reported that the *IGK* assay help maximize detection [27, 28] in approximately 6–7% of diffuse large B cell lymphomas and that plasma cell neoplasms remain without a detectable clones when only *IGH* is targeted. Interestingly, Rustad et al. found that the success rate of the *IGK* assay in patients with lambda-restricted MM (72%) was significantly higher than that in patients with kappa-restricted MM (45%) [9]. This was attributed to lambda light chain expression requiring a functional lambda light chain gene, which happens only after both *IGK* alleles have been inactivated through the rearrangement of the KDE region to partner with either V_k_ in naïve *IGK* alleles or with the intronic region between J_k_ and C_k_. A similar phenomenon was observed in our study in that clonal *IGK* rearrangements were observed in 67.7% of samples from patients with kappa-restricted MM and 95.5% of those with lambda-restricted MM, demonstrating the benefit of *IGK* testing in addition to *IGH* in such patients.

The presence and extent of clonal rearrangement and the proportion of BM plasma cells as a proportion of all BM lymphoid cells were not significantly correlated in our study. There was a huge overlap in BM plasma cells as a proportion of all BM lymphoid cells between patients with clonal *IGH* and/or *IGK* rearrangement and those without any statistical significance (median [range]: 76.4 [28.5–100.0%] and 64.5 [32.6–92.5%]). Most previous studies did not investigate the association between clonal *IG* gene rearrangement and the proportion of BM plasma cells. Rustad et al. suggested that low tumor cell content was the main reason for the failure to identify a clonal V(D)J sequence; while samples with more than 5% plasma cells had a 97% clonality detection rate, those with less than 5% plasma cells had a detection rate of ~70% [9]. However, they also acknowledged an extensive overlap between groups with and without clonal rearrangement, which was consistent with our own findings. In a study of B-cell neoplasm samples that included MM, Arcila et al. reported that some samples with 5–68% tumor content remained nonclonal when tested using IGH primer sets [4], suggesting that the tumor cell proportion itself does not necessarily influence the detection of clonal rearrangement. Similar to the BIOMED-2/EuroClonality approach, NGS-based clonality assessment also relies on an initial multiplex PCR step, which could still be hampered by somatic hypermutation and specific polymorphisms that might prevent primer annealing [5]. Indeed, studies aimed at validating the limit of detection and linearity of NGS-based *IG* gene rearrangement tests found that clonal rearrangements could be traced to dilutions as low as 2.5% [4, 5]. Additional studies with a larger number of samples and with variable plasma cell percentages would be helpful for clarifying the impact of the plasma cell proportion on clonality detection.

The repertoire of *IGH* and *IGK* gene usage in Korean patients with MM has not been reported to date; although data from few Western studies exist [7, 8, 24], there are no data from studies on Asian patients. Notably, a comparison between our findings and those of previous Western studies revealed that *IGHV3-9*, *IGHV4-31*, and *IGHD3-3* were common in Korean patients with MM but not in Western patients; no *IGK* rearrangement was found to be more frequent in Korean patients than in Western counterparts, particularly among those with kappa-restricted MM. Moreover, the single *IGKV-J* rearrangement type was more frequent in Western patients with kappa-restricted MM than in Korean counterparts; *IGKV4-1* was more frequent in Western than in Korean patients regardless of light chain type. These data suggested the existence of ethnicity-based differences in disease risk and manifestation [7, 8, 24]. Otherwise, the *IGH* and *IGK* repertoire distribution in our study showed a similar pattern to those of Western studies [7, 8, 24]. Ferrero et al. found that *IGH* gene usage in MM was similar to that of normal plasma cells [7]. Hadzidimitriou et al. reported that the *IGH* repertoire followed a similar pattern to that of the normal repertoire [8]. They also reported that the *IGHV4-34* gene was rarely observed in MM, which was consistent with older *IG* profiling studies in patients with this disease [29–31]; in contrast, the *IGHV4-34* gene is common in B-lymphoid malignancies, particularly chronic lymphocytic leukemia [8]. Medina et al. reported that gene selection was biased in MM, with a significant overrepresentation of *IGHV3*, *IGHD2*, and *IGHD3*, as well as of the *IGHJ4* gene group, compared to the normal B-cell repertoire [24]. *IGKV-J* rearrangement was the most common (8 of 14, 57.1%), followed by *KDE* (5 of 14, 35.7%). *IGKJ4* was the most predominant (5 of 14, 35.7%), and the distribution of the *IGK* gene usage in our study was similar to those found in previous studies except that the most frequent *IGK* gene observed in the only available Western study that analyzed the *IGK* repertoire, *IGKV4-1*, was not observed among our patients [8]. This also could be attributed to ethnicity-based or risk group differences. Further studies involving a larger number of Korean patients with MM would be helpful for confirming our observations.

The strengths of our study include that (i) we investigated the applicability of the NGS-based *IGH*/*IGK* rearrangement test for the detection of clonality in Asians with MM for the first time, (ii) we analyzed the profiles of *IGH* and *IGK* gene usage in Asians with MM and compared them with those from previous Western studies for the first time, and (iii) we investigated the association between clonal *IGH*/*IGK* rearrangement and the proportion of BM lymphoid cells that were plasma cells. Nevertheless, there were a few limitations, some aforementioned, as follows: (i) the number of samples was relatively small, (ii) the majority of samples were from high-risk patients with a relatively high percentage of BM plasma cells, and (iii) *IGH* FR2 and *IGH* FR3 tests were not performed.

In summary, we showed that clonal *IGH* and/or *IGK* rearrangements were detected in 88.1% of Koreans with MM at baseline, indicating that such tests are applicable to most Korean patients with this disease to determine clonality and to monitor those under treatment. The profile of *IGH* and *IGK* gene usage in Koreans with MM was similar to those found in Western MM studies. However, *IGHV3-9*, *IGHV4-31*, and *IGHD3-3* were overrepresented among Korean patients, whereas *IGKV4-1* was underrepresented. No *IGK* rearrangement was found to be more common in Koreans with kappa-restricted MM, although the *IGKV-J* rearrangement type was more frequent in Western counterparts. This implied the existence of certain ethnicity-based differences in the characteristics of patients with MM.

## Supporting information

S1 File (DOCX)

S2 File (DOCX)

S3 File (XLSX)
