# Supplementary figures and images for: Immunoglobulin gene rearrangement in Koreans with multiple myeloma: Clonality assessment and repertoire analysis using next-generation sequencing
Source: PLoS One. 2021 Jun 24;16(6):e0253541. doi: 10.1371/journal.pone.0253541 (PMC8224885; doi:10.1371/journal.pone.0253541)

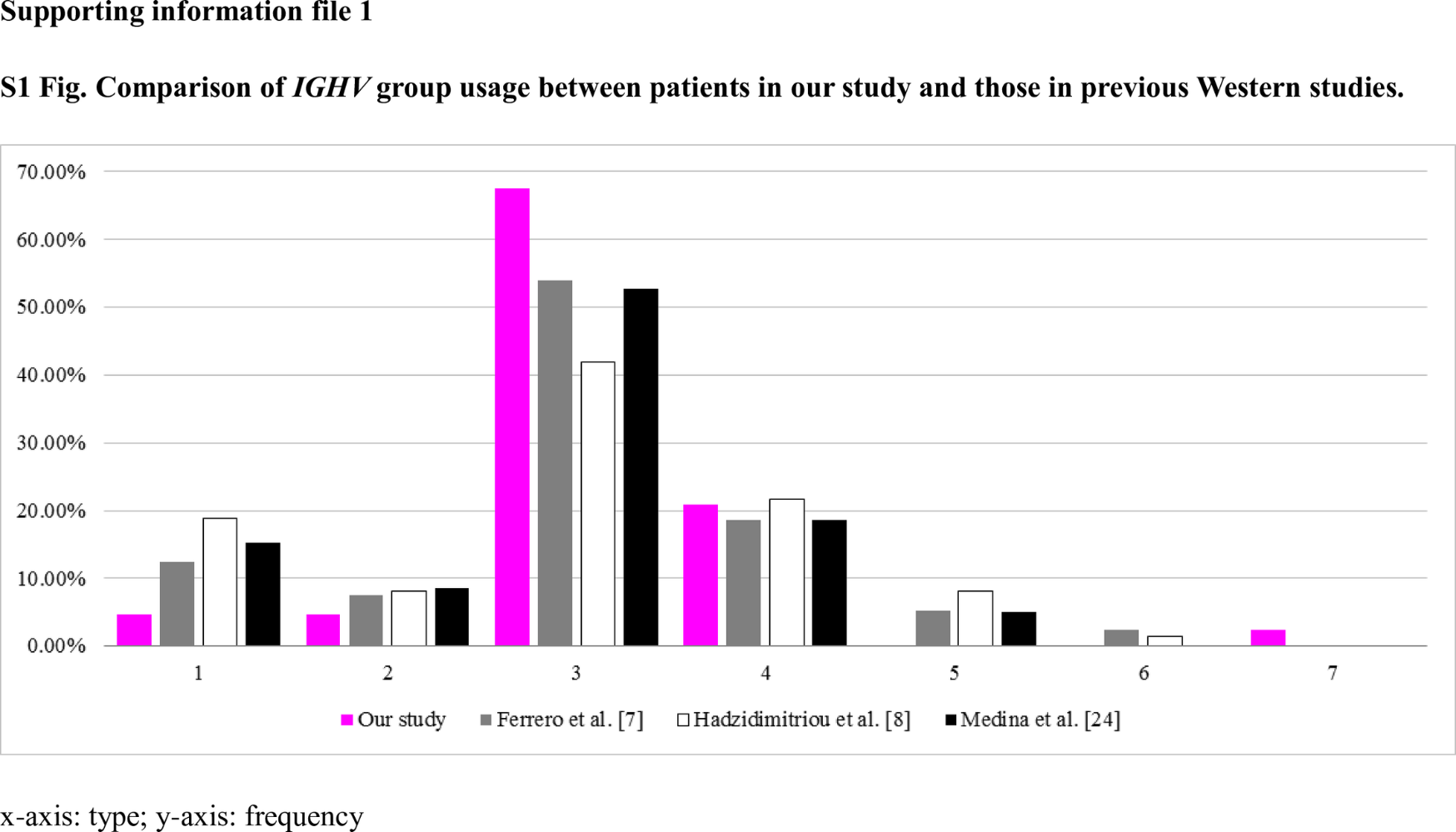


**
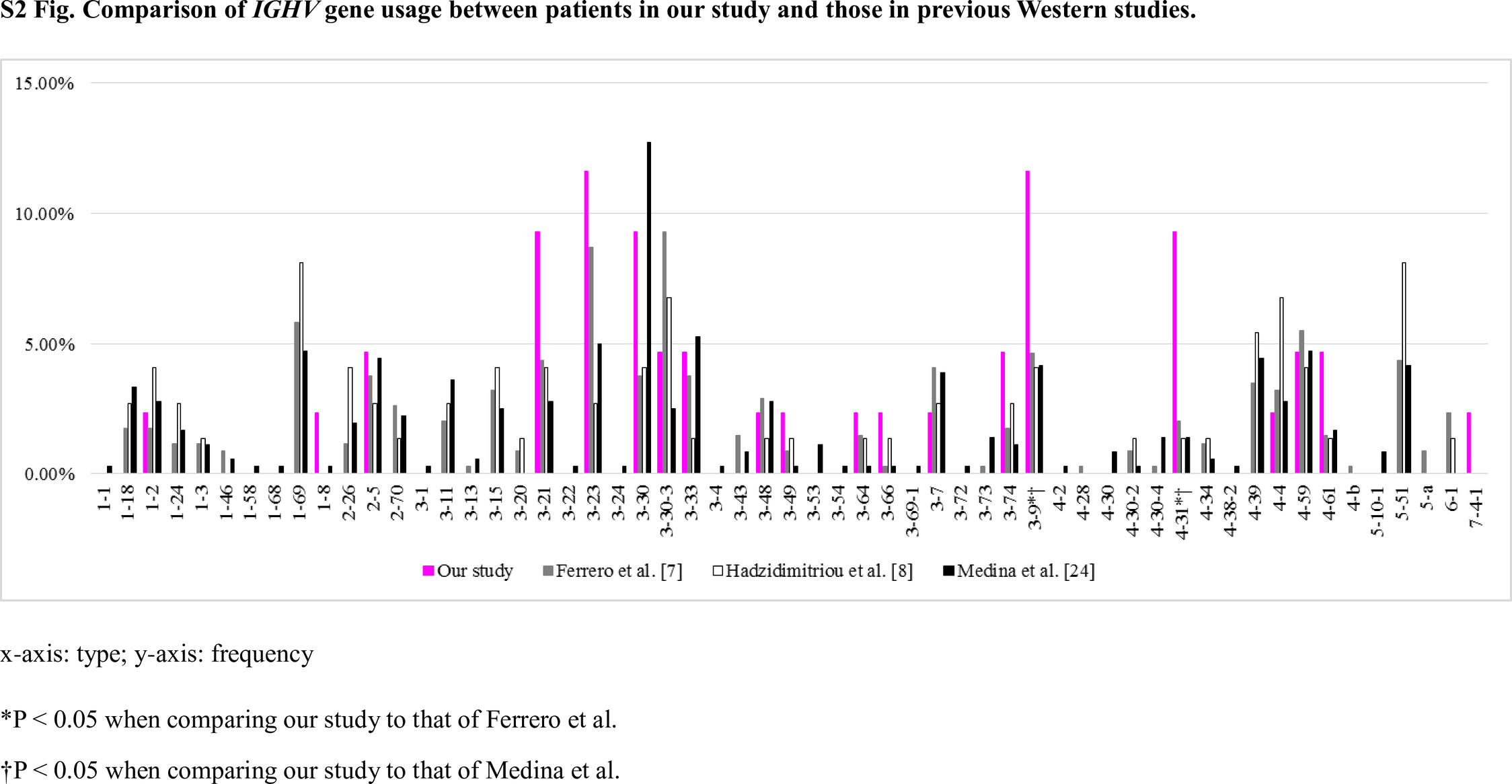
**

**
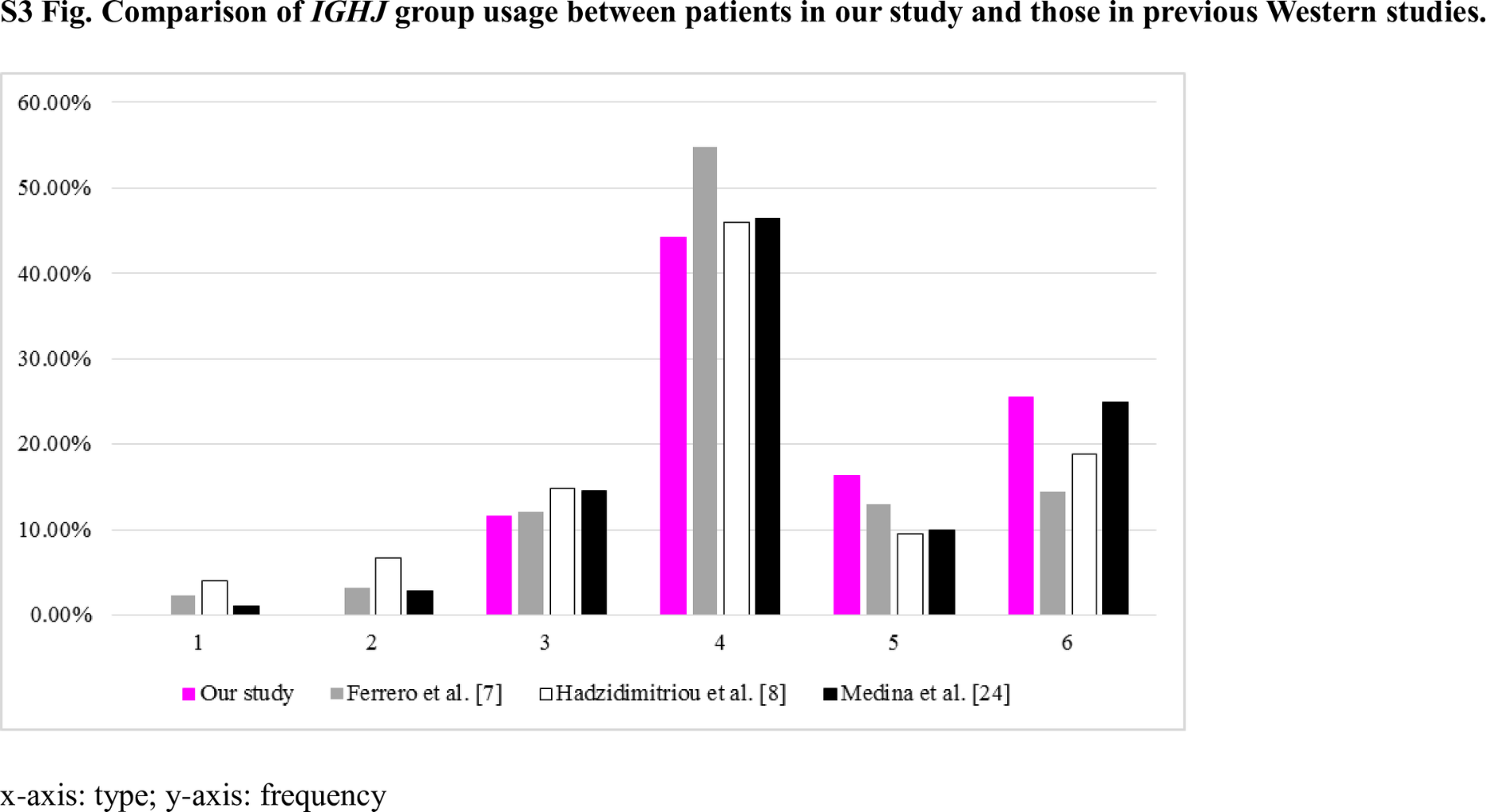
**

**
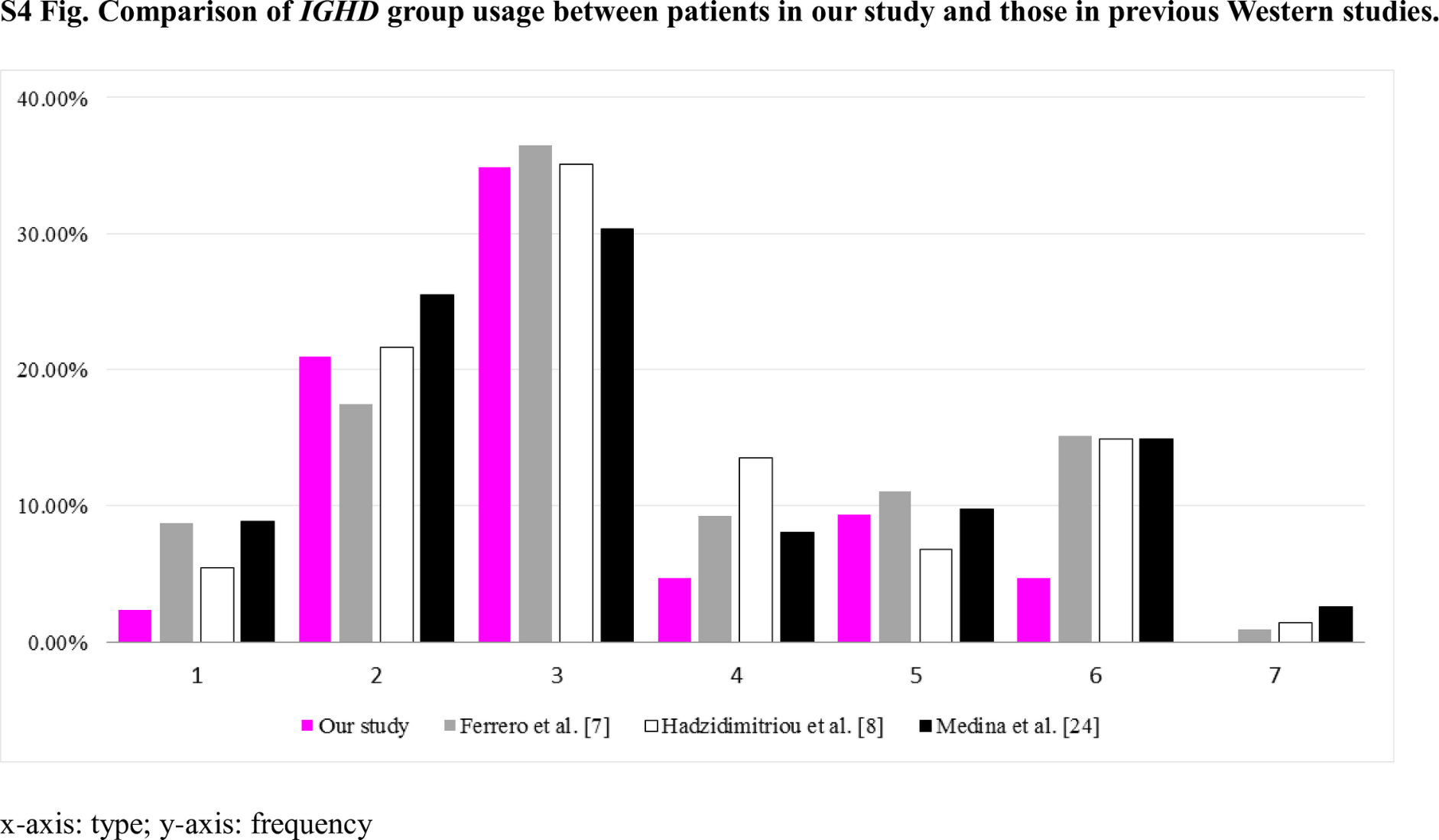
**

**
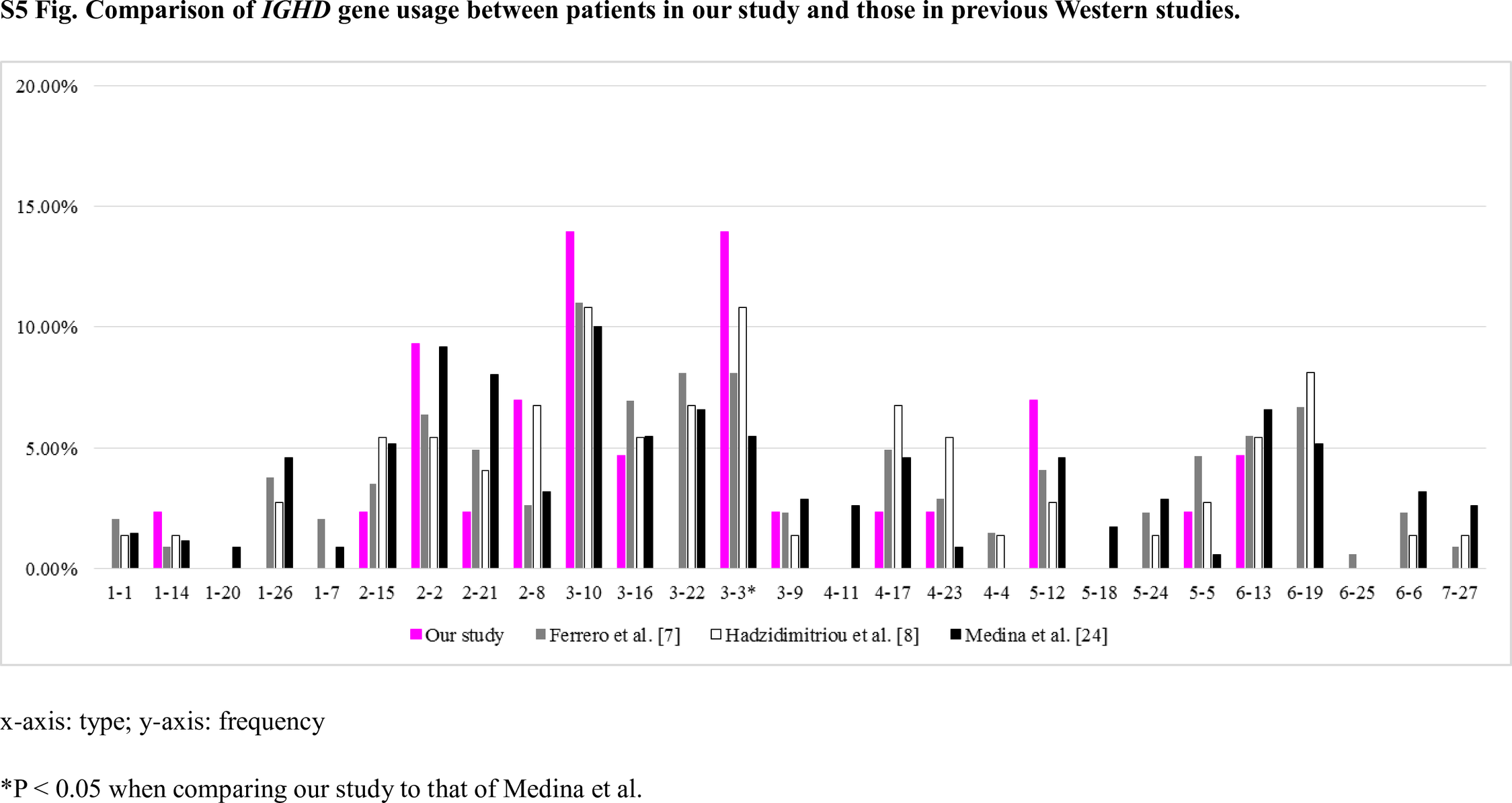
**

**
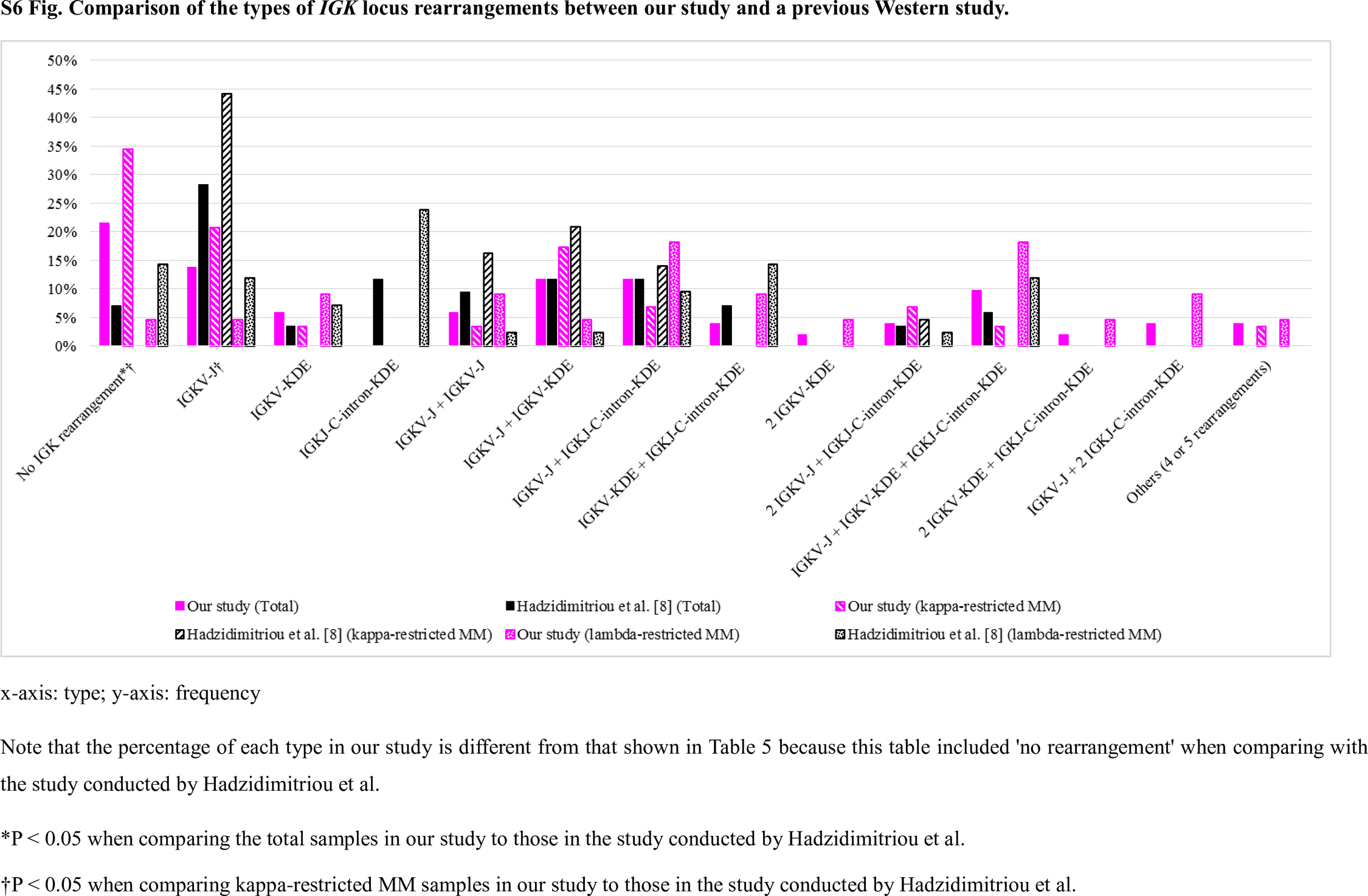
**

**
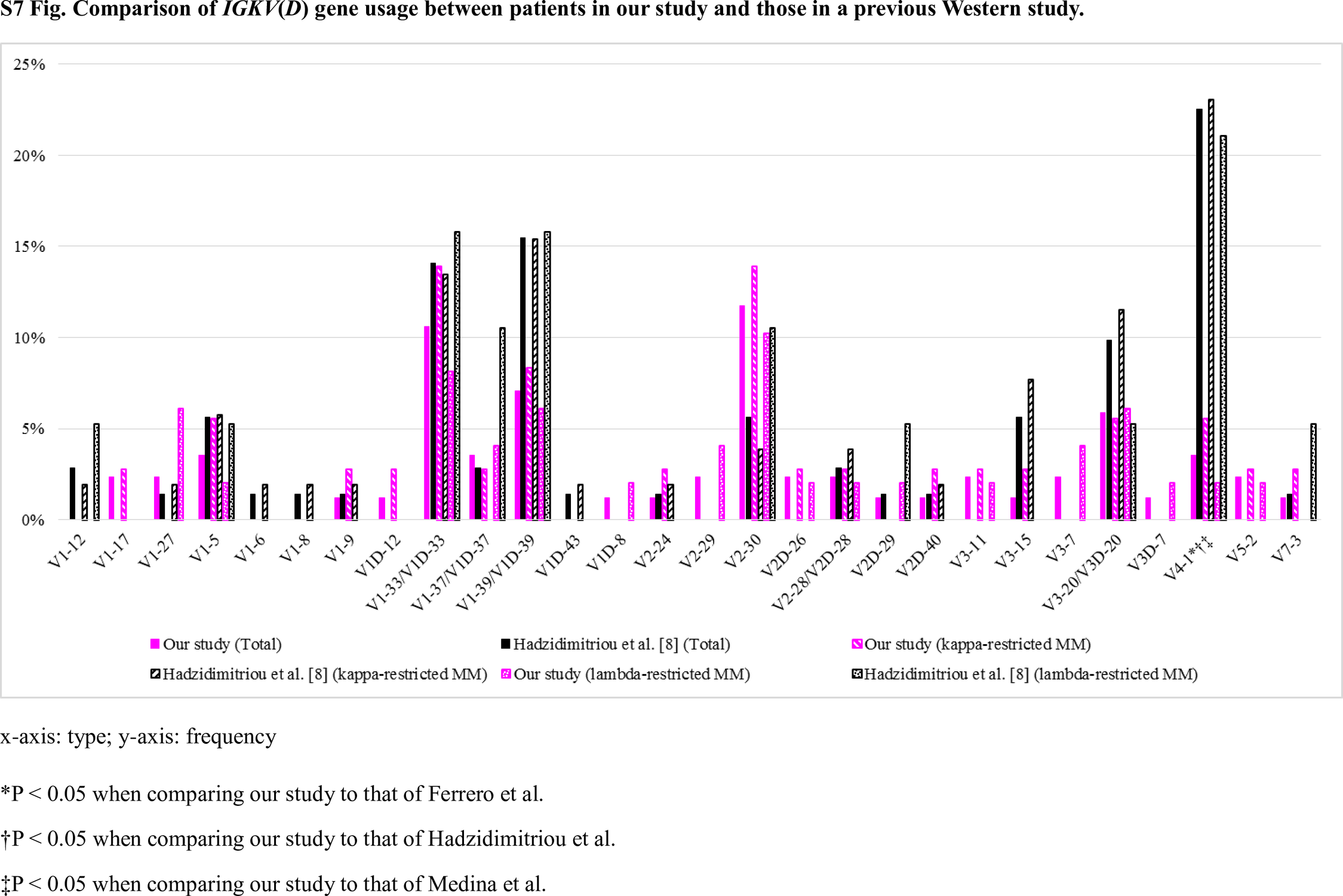
**

Supplement: S1 File — (DOCX) [file pone.0253541.s001.docx]
