# Supplementary material for: Immunoglobulin gene rearrangement in Koreans with multiple myeloma: Clonality assessment and repertoire analysis using next-generation sequencing
Source: PLoS One. 2021 Jun 24;16(6):e0253541. doi: 10.1371/journal.pone.0253541 (PMC8224885; doi:10.1371/journal.pone.0253541)
Supplement: S2 File — (DOCX) [file pone.0253541.s002.docx]

**S2 File**

**S1 Table. Comparison of the *IGHV* group usage between our study and previous Western studies.**

|  |  |  |  |  |
| --- | --- | --- | --- | --- |
| Type | Our study | Ferrero et al. [7] | Hadzidimitriou et al. [8] | Medina et al. [24] |
|  | n = 43 | n = 345 | n = 74 | n = 362 |
| 1 | 4.65% | 12.46% | 18.92% | 15.19% |
| 2 | 4.65% | 7.54% | 8.11% | 8.56% |
| 3 | 67.44% | 53.91% | 41.89% | 52.76% |
| 4 | 20.93% | 18.55% | 21.62% | 18.51% |
| 5 |  | 5.22% | 8.11% | 4.97% |
| 6 |  | 2.32% | 1.35% |  |
| 7 | 2.33% |  |  |  |

**S2 Table. Comparison of the *IGHV* gene usage between our study and previous Western studies.**

|  |  |  |  |  |
| --- | --- | --- | --- | --- |
| Type | Our study | Ferrero et al. [7] | Hadzidimitriou et al. [8] | Medina et al. [24] |
|  | n = 43 | n = 345 | n = 74 | n = 362 |
| 1-1 |  |  |  | 0.28% |
| 1-18 |  | 1.74% | 2.70% | 3.31% |
| 1-2 | 2.33% | 1.74% | 4.05% | 2.76% |
| 1-24 |  | 1.16% | 2.70% | 1.66% |
| 1-3 |  | 1.16% | 1.35% | 1.10% |
| 1-46 |  | 0.87% |  | 0.55% |
| 1-58 |  |  |  | 0.28% |
| 1-68 |  |  |  | 0.28% |
| 1-69 |  | 5.80% | 8.11% | 4.70% |
| 1-8 | 2.33% |  |  | 0.28% |
| 2-26 |  | 1.16% | 4.05% | 1.93% |
| 2-5 | 4.65% | 3.77% | 2.70% | 4.42% |
| 2-70 |  | 2.61% | 1.35% | 2.21% |
| 3-1 |  |  |  | 0.28% |
| 3-11 |  | 2.03% | 2.70% | 3.59% |
| 3-13 |  | 0.29% |  | 0.55% |
| 3-15 |  | 3.19% | 4.05% | 2.49% |
| 3-20 |  | 0.87% | 1.35% |  |
| 3-21 | 9.30% | 4.35% | 4.05% | 2.76% |
| 3-22 |  |  |  | 0.28% |
| 3-23 | 11.63% | 8.70% | 2.70% | 4.97% |
| 3-24 |  |  |  | 0.28% |
| 3-30 | 9.30% | 3.77% | 4.05% | 12.71% |
| 3-30-3 | 4.65% | 9.28% | 6.76% | 2.49% |
| 3-33 | 4.65% | 3.77% | 1.35% | 5.25% |
| 3-4 |  |  |  | 0.28% |
| 3-43 |  | 1.45% |  | 0.83% |
| 3-48 | 2.33% | 2.90% | 1.35% | 2.76% |
| 3-49 | 2.33% | 0.87% | 1.35% | 0.28% |
| 3-53 |  |  |  | 1.10% |
| 3-54 |  |  |  | 0.28% |
| 3-64 | 2.33% | 1.45% | 1.35% | 0.28% |
| 3-66 | 2.33% | 0.29% | 1.35% | 0.28% |
| 3-69-1 |  |  |  | 0.28% |
| 3-7 | 2.33% | 4.06% | 2.70% | 3.87% |
| 3-72 |  |  |  | 0.28% |
| 3-73 |  | 0.29% |  | 1.38% |
| 3-74 | 4.65% | 1.74% | 2.70% | 1.10% |
| 3-9*† | 11.63% | 4.64% | 4.05% | 4.14% |
| 4-2 |  |  |  | 0.28% |
| 4-28 |  | 0.29% |  |  |
| 4-30 |  |  |  | 0.83% |
| 4-30-2 |  | 0.87% | 1.35% | 0.28% |
| 4-30-4 |  | 0.29% |  | 1.38% |
| 4-31*† | 9.30% | 2.03% | 1.35% | 1.38% |
| 4-34 |  | 1.16% | 1.35% | 0.55% |
| 4-38-2 |  |  |  | 0.28% |
| 4-39 |  | 3.48% | 5.41% | 4.42% |
| 4-4 | 2.33% | 3.19% | 6.76% | 2.76% |
| 4-59 | 4.65% | 5.51% | 4.05% | 4.70% |
| 4-61 | 4.65% | 1.45% | 1.35% | 1.66% |
| 4-b |  | 0.29% |  |  |
| 5-10-1 |  |  |  | 0.83% |
| 5-51 |  | 4.35% | 8.11% | 4.14% |
| 5-a |  | 0.87% |  |  |
| 6-1 |  | 2.32% | 1.35% |  |
| 7-4-1 | 2.33% |  |  |  |

*P < 0.05 when comparing our study to that of Ferrero et al.

†P < 0.05 when comparing our study to that of Medina et al.

**S3 Table. Comparison of *IGHJ* group usage between our study and previous Western studies.**

|  |  |  |  |  |
| --- | --- | --- | --- | --- |
| Type | Our study | Ferrero et al. [7] | Hadzidimitriou et al. [8] | Medina et al. [24] |
|  | n = 43 | n = 345 | n = 74 | n = 349 |
| 1 |  | 2.32% | 4.05% | 1.15% |
| 2 |  | 3.19% | 6.76% | 2.87% |
| 3 | 11.63% | 12.17% | 14.86% | 14.61% |
| 4 | 44.19% | 54.78% | 45.95% | 46.42% |
| 5 | 16.28% | 13.04% | 9.46% | 10.03% |
| 6 | 25.58% | 14.49% | 18.92% | 24.93% |
| No J Gene | 2.33% |  |  |  |

**S4 Table. Comparison of *IGHD* group usage between our study and previous Western studies.**

|  |  |  |  |  |
| --- | --- | --- | --- | --- |
| Type | Our study | Ferrero et al. [7] | Hadzidimitriou et al. [8] | Medina et al. [24] |
|  | n = 43 | n = 345 | n = 74 | n = 349 |
| 1 | 2.33% | 8.70% | 5.41% | 8.88% |
| 2 | 20.93% | 17.39% | 21.62% | 25.50% |
| 3 | 34.88% | 36.52% | 35.14% | 30.37% |
| 4 | 4.65% | 9.28% | 13.51% | 8.02% |
| 5 | 9.30% | 11.01% | 6.76% | 9.74% |
| 6 | 4.65% | 15.07% | 14.86% | 14.90% |
| 7 |  | 0.87% | 1.35% | 2.58% |
| Unknown | 11.63% |  |  |  |
| Not found | 11.63% | 1.16% | 1.35% |  |

**S5 Table. Comparison of *IGHD* gene usage between our study and previous Western studies.**

|  |  |  |  |  |
| --- | --- | --- | --- | --- |
| Type | Our study | Ferrero et al. [7] | Hadzidimitriou et al. [8] | Medina et al. [24] |
|  | n = 43 | n = 345 | n = 74 | n = 349 |
| 1-1 |  | 2.03% | 1.35% | 1.43% |
| 1-14 | 2.33% | 0.87% | 1.35% | 1.15% |
| 1-20 |  |  |  | 0.86% |
| 1-26 |  | 3.77% | 2.70% | 4.58% |
| 1-7 |  | 2.03% |  | 0.86% |
| 2-15 | 2.33% | 3.48% | 5.41% | 5.16% |
| 2-2 | 9.30% | 6.38% | 5.41% | 9.17% |
| 2-21 | 2.33% | 4.93% | 4.05% | 8.02% |
| 2-8 | 6.98% | 2.61% | 6.76% | 3.15% |
| 3-10 | 13.95% | 11.01% | 10.81% | 10.03% |
| 3-16 | 4.65% | 6.96% | 5.41% | 5.44% |
| 3-22 |  | 8.12% | 6.76% | 6.59% |
| 3-3* | 13.95% | 8.12% | 10.81% | 5.44% |
| 3-9 | 2.33% | 2.32% | 1.35% | 2.87% |
| 4-11 |  |  |  | 2.58% |
| 4-17 | 2.33% | 4.93% | 6.76% | 4.58% |
| 4-23 | 2.33% | 2.90% | 5.41% | 0.86% |
| 4-4 |  | 1.45% | 1.35% | 0.00% |
| 5-12 | 6.98% | 4.06% | 2.70% | 4.58% |
| 5-18 |  |  |  | 1.72% |
| 5-24 |  | 2.32% | 1.35% | 2.87% |
| 5-5 | 2.33% | 4.64% | 2.70% | 0.57% |
| 6-13 | 4.65% | 5.51% | 5.41% | 6.59% |
| 6-19 |  | 6.67% | 8.11% | 5.16% |
| 6-25 |  | 0.58% |  | 0.00% |
| 6-6 |  | 2.32% | 1.35% | 3.15% |
| 7-27 |  | 0.87% | 1.35% | 2.58% |
| Unknown | 11.63% |  |  |  |
| Not found | 11.63% | 1.16% | 1.35% |  |

*P < 0.05 when comparing our study to that of Medina et al.

**S6 Table. Comparison of the type of *IGK* locus rearrangements between our study and a previous Western study.**

|  |  |  |  | |  |  | |  |
| --- | --- | --- | --- | --- | --- | --- | --- | --- |
| Type | Total | | Kappa-restricted MM | | | Lambda-restricted MM | | |
|  | Our study | Hadzidimitriou et al. [8] | Our study | Hadzidimitriou et al. [8] | | Our study | Hadzidimitriou et al. [8] | |
|  | n = 51 | n = 85 | n = 29 | n = 43 | | n = 22 | n = 42 | |
| No *IGK* rearrangement*† | 21.57% | 7.06% | 34.48% | 0.00% | | 4.55% | 14.29% | |
| *IGKV-J*† | 13.73% | 28.24% | 20.69% | 44.19% | | 4.55% | 11.90% | |
| *IGKV-KDE* | 5.88% | 3.53% | 3.45% | 0.00% | | 9.09% | 7.14% | |
| *IGKJ-C-intron-KDE* | 0.00% | 11.76% | 0.00% | 0.00% | | 0.00% | 23.81% | |
| *IGKV-J + IGKV-J* | 5.88% | 9.41% | 3.45% | 16.28% | | 9.09% | 2.38% | |
| *IGKV-J + IGKV-KDE* | 11.76% | 11.76% | 17.24% | 20.93% | | 4.55% | 2.38% | |
| *IGKV-J + IGKJ-C-intron-KDE* | 11.76% | 11.76% | 6.90% | 13.95% | | 18.18% | 9.52% | |
| *IGKV-KDE + IGKJ-C-intron-KDE* | 3.92% | 7.06% | 0.00% | 0.00% | | 9.09% | 14.29% | |
| 2 *IGKV-KDE* | 1.96% | 0.00% | 0.00% | 0.00% | | 4.55% | 0.00% | |
| 2 *IGKV-J + IGKJ-C-intron-KDE* | 3.92% | 3.53% | 6.90% | 4.65% | | 0.00% | 2.38% | |
| *IGKV-J + IGKV-KDE + IGKJ-C-intron-KDE* | 9.80% | 5.88% | 3.45% | 0.00% | | 18.18% | 11.90% | |
| 2 *IGKV-KDE + IGKJ-C-intron-KDE* | 1.96% | 0.00% | 0.00% | 0.00% | | 4.55% | 0.00% | |
| *IGKV-J +* 2 *IGKJ-C-intron-KDE* | 3.92% | 0.00% | 0.00% | 0.00% | | 9.09% | 0.00% | |
| Others (4 or 5 rearrangements) | 3.92% | 0.00% | 3.45% | 0.00% | | 4.55% | 0.00% | |

Note that the percentage of each type in our study is different from that of Table 5 because this table included 'no rearrangement' when comparing data with those in Hadzidimitriou et al.'s study.

*P < 0.05 when comparing the total samples in our study with those in Hadzidimitriou et al.'s study

†P < 0.05 when comparing the kappa-restricted MM samples in our study with those in Hadzidimitriou et al.'s study

**S7 Table. Comparison of *IGKV*(*D*) usage between our study and a previous Western study.**

|  |  |  |  | |  |  | |  |
| --- | --- | --- | --- | --- | --- | --- | --- | --- |
| Type | Total | | Kappa-restricted MM | | | Lambda-restricted MM | | |
|  | Our study | Hadzidimitriou et al. [8] | Our study | Hadzidimitriou et al. [8] | | Our study | Hadzidimitriou et al. [8] | |
|  | n = 85 | n = 71 | n = 36 | n = 52 | | n = 49 | n = 19 | |
| *V1-12* |  | 2.82% |  | 1.92% | |  | 5.26% | |
| *V1-17* | 2.35% |  | 2.78% |  | |  |  | |
| *V1-27* | 2.35% | 1.41% |  | 1.92% | | 6.12% |  | |
| *V1-5* | 3.53% | 5.63% | 5.56% | 5.77% | | 2.04% | 5.26% | |
| *V1-6* |  | 1.41% |  | 1.92% | |  |  | |
| *V1-8* |  | 1.41% |  | 1.92% | |  |  | |
| *V1-9* | 1.18% | 1.41% | 2.78% | 1.92% | |  |  | |
| *V1D-12* | 1.18% |  | 2.78% |  | |  |  | |
| *V1-33/V1D-33* | 10.59% | 14.08% | 13.89% | 13.46% | | 8.16% | 15.79% | |
| *V1-37/V1D-37* | 3.53% | 2.82% | 2.78% |  | | 4.08% | 10.53% | |
| *V1-39/V1D-39* | 7.06% | 15.49% | 8.33% | 15.38% | | 6.12% | 15.79% | |
| *V1D-43* |  | 1.41% |  | 1.92% | |  |  | |
| *V1D-8* | 1.18% |  |  |  | | 2.04% |  | |
| *V2-24* | 1.18% | 1.41% | 2.78% | 1.92% | |  |  | |
| *V2-29* | 2.35% |  |  |  | | 4.08% |  | |
| *V2-30* | 11.76% | 5.63% | 13.89% | 3.85% | | 10.20% | 10.53% | |
| *V2D-26* | 2.35% |  | 2.78% |  | | 2.04% |  | |
| *V2-28/V2D-28* | 2.35% | 2.82% | 2.78% | 3.85% | | 2.04% |  | |
| *V2D-29* | 1.18% | 1.41% |  |  | | 2.04% | 5.26% | |
| *V2D-40* | 1.18% | 1.41% | 2.78% | 1.92% | |  |  | |
| *V3-11* | 2.35% |  | 2.78% |  | | 2.04% |  | |
| *V3-15* | 1.18% | 5.63% | 2.78% | 7.69% | |  |  | |
| *V3-7* | 2.35% |  |  |  | | 4.08% |  | |
| *V3-20/V3D-20* | 5.88% | 9.86% | 5.56% | 11.54% | | 6.12% | 5.26% | |
| *V3D-7* | 1.18% |  |  |  | | 2.04% |  | |
| *V4-1**†‡ | 3.53% | 22.54% | 5.56% | 23.08% | | 2.04% | 21.05% | |
| *V5-2* | 2.35% |  | 2.78% |  | | 2.04% |  | |
| *V7-3* | 1.18% | 1.41% | 2.78% |  | |  | 5.26% | |
| *Intron-KDE* | 24.71% |  | 13.89% |  | | 32.65% |  | |

*P < 0.05 when comparing our study to that of Ferrero et al.

†P < 0.05 when comparing our study to that of Hadzidimitriou et al.

‡P < 0.05 when comparing our study to that of Medina et al.
